# Effects of early-life amino acids supplementation on fish responses to a thermal challenge

**DOI:** 10.1007/s00360-024-01581-1

**Published:** 2024-09-13

**Authors:** Carmen Navarro-Guillén, Ismael Jerez-Cepa, André Lopes, Juan Miguel Mancera, Sofia Engrola

**Affiliations:** 1 Centre of Marine Sciences (CCMAR/CIMAR LA), Universidade do Algarve, Campus de Gambelas, Faro, 8005-139 Portugal; 2 Department of Biology, Faculty of Marine and Environmental Sciences, Institute of Marine Research (INMAR), Universidad de Cádiz, CEI·MAR, Puerto Real, Cádiz, Spain; 3 Present Address: Departmento de Biología Marina y Acuicultura, Instituto de Ciencias Marinas de Andalucía (ICMAN-CSIC), Puerto Real, Cádiz, Spain

**Keywords:** Amino acids, Early nutritional programming, Fish thermal adaptation, Water temperature, Zebrafish larvae

## Abstract

Nutritional programming is a promising concept for promoting metabolic adaptation of fish to challenging conditions, such as the increase in water temperature. The present work evaluates in ovo arginine or glutamine supplementation as enhancers of zebrafish metabolic or absorptive capacity, respectively, at optimum (28 ºC) and challenging temperatures (32 ºC) in the long-term. Growth performance, free amino acids profile, methylation index and the activity levels of digestive and intermediary metabolism enzymes were analysed to assess the metabolic plasticity induced by an early nutritional intervention. Temperature affected fish larvae growth performance. At the end of the experimental period 28 ºC-fish showed higher dry weight than 32 ºC-fish. The effects of the early supplementation were reflected in the larval free amino acids profile at the end of the experiment. Higher methylation potential was observed in the ARG-fish. In ovo amino acid supplementation modulated the metabolic response in zebrafish larvae, however, the magnitude of this effect differed according to the amino acid and the temperature. Overall, arginine supplementation enhanced carbohydrates metabolism at 32 ºC. In conclusion, the present work suggests that in ovo arginine supplementation may promote a better adaptive response to higher temperatures.

**Supplementary Information:**

The online version contains supplementary material available at 10.1007/s00360-024-01581-1.

## Introduction

The effects of climate change, such as the increase in water temperature, is challenging aquatic organisms’ resilience. From a food web context, the upcoming long-term scenario will affect the future size-structure, biomass production and potential yields of marine and freshwater fish (Mugwanya et al. 2022; Lindmark et al. 2022). Hence, progress to attain climate resilient fish will advance by promoting the ability of animals to recover and adapt to environmental changes (Abisha et al. 2022). Identifying strategies that enhances fish ability to cope with the environmental challenges will have a massive impact on fish growth and robustness.

As ectotherms, fish body temperature is equivalent to the surrounding water temperature. Elevated temperatures impact fish physiology and behaviour by heightening metabolic demands and influencing nutrient utilization and growth (Campos et al. 2013a, b; Boltaña et al. 2017; Yúfera et al. 2019; Navarro-Guillén et al. 2023). Impacts on fish physiology due to global warming are expected in the long-term to decrease fish maximum body mass (Cheung et al. 2013). At the optimal temperature of a species, it is suggested that a lower cost of maintenance is associated with a greater energy allocation for growth; conversely, elevated temperatures increase metabolic demand, leading to a reduced allocation of energy for growth (Killen 2014). A mandatory trade-off between maintenance and growth will be critical to fish acclimate to any abiotic change. In this regard, exploring nutritional strategies to foster climate-resilient farmed fish is pivotal to establish a robust food supply chain under an environmentally changing scenario. Early programming is defined as an event in early life that exerts long-term effects on the individual as result of adaptive changes at the molecular, cellular, and biochemical levels (Lucas 1998). This physiological “setting” is driven by an early stimulus or insult at a critical point. In fish, the embryonic development or larval stage has been described as one of the most responsive periods to metabolic programming (Engrola et al. 2018). An in ovo stimulus of ghrelin in zebrafish (*Danio rerio*) showed that larvae present an increased swimming activity, suggesting that the peptide may have a relevant role in foraging activity (Navarro-Guillén et al. 2019). Rocha et al. (2016a) assessed the effect of early nutritional programming through exogenous feeding on specific metabolic pathways modulation in gilthead seabream (*Sparus aurata*) and found that a dietary glucose stimulus promoted changes in the larval metabolism and utilization of carbohydrates, that were still visible at juvenile stage (Rocha et al. 2016b). Feeding Senegalese sole (*Solea senegalensis*) at mouth opening a low dietary protein complexity promoted in postlarvae an upregulation in genes encoding for de novo DNA methyltransferases suggesting a potential programming for regulation of myogenesis (Canada et al. 2018). Although nutritional strategies at first feeding exerted positive long-lasting effects, in ovo exposure to a nutritional stimulus may have several advantages. Firstly, the developmental window in which the stimulus is applied. During embryonic development, physiological pathways are being formed while fish rely exclusively on yolk nutrients, this may increase susceptibility to nutritional modifications than later (Navarro-Guillén et al. 2019). In addition, sonophoresis, the technique applied in the present study based on low-frequency ultrasounds, allowed nutritional modulation in a batch of eggs in a single intervention.

The role of specific amino acids in critical metabolic pathways that modulates fish growth, health, or stress response is widely recognized (Rønnestad et al. 2003; Conceição et al. 2012; Andersen et al. 2016; Canada et al. 2016; Herrera et al. 2019; Li et al. 2021). The amino acids are divided into two categories based on their absolute or relative rates of synthesis in vivo: (i) indispensable amino acids (IAA), which are needed by the organism because they cannot be synthesized or the synthesis is not rapid enough to satisfy the physiological demands and, (ii) dispensable amino acids (DAA) which can be synthesized in vivo (Lall and Anderson 2005). Fish seem to have the capacity to spare IAA at the expense of DAA since early stages of development (Conceição et al. 2003).

Arginine is an indispensable amino acid that can promote fish growth through the enhancement of fish robustness, since it is one of the most important functional amino acids. Dietary arginine exerted positive effects on the antioxidant, stress and immune responses in different fish species (Oehme et al. 2010; Costas et al. 2011; Pohlenz et al. 2014; Azeredo et al. 2020; Hoseini et al. 2020; Ramos-Pinto et al. 2020). In addition, dietary arginine promoted fish resistance to chronic stress, such as hypoxia (Costas et al. 2013). In broiler chickens, dietary arginine supplementation partially counterbalanced the effects of heat stress on energy homeostasis (Brugaletta et al. 2023). Glutamine, a dispensable amino acid, fostered fish growth through the promotion of intestinal development and functionality in several species and developmental stages (Cheng et al. 2011; Li et al. 2020; Macêdo et al. 2021; Palomino Ramos et al. 2022; Carvalho et al. 2023). In Jian carp (*Cyprinus carpio* var. Jian) H_2_O_2_-induced enterocyte injury was restored after culturing the cells in media with glutamine (Hu et al. 2014). Under heat stress, dietary supplementation of glutamine effectively mitigated its deleterious effects by increasing growth performances in broilers chickens (Ncho et al. 2023). The impact of an in ovo stimulus with arginine or glutamine was assessed in zebrafish larvae in the long-term (Navarro-Guillén et al. 2021). At the end of the experiment, larvae originated from eggs supplemented with glutamine presented higher growth performance and better digestive capacity through a positive modulation of gut microbiota. The role of amino acids supplementation has been studied in several fish species, however their potential when supplemented before mouth opening is still unforeseen.

The aim of this study was to evaluate how an early in ovo supplementation of specific amino acids could assist fish in dealing with an environmental stressor, such as high temperature. For that purpose, zebrafish eggs were supplemented with: (A) an indispensable amino acid, arginine, as an enhancer of the systemic metabolism or, (B) a dispensable amino acid, glutamine, as a promoter of fish digestive and absorptive capacities. In ovo supplementation was performed using sonophoresis technique and, after supplementation, fish were reared at optimum temperature (28 ºC) or at challenging temperature (32 ºC). Growth performance, free amino acid profile, methylation potential and the metabolic responses to a chronic thermal stress were analysed.

## Materials and methods

In the present study zebrafish was used as fish model species with translational applications to other fish, both farmed and wild species, since it has been successfully used as an animal model for the study of different issues regarding fish physiological responses to the environment for more than two decades (Piferrer and Ribas 2020).

### Experimental design

The objective of this experiment was to investigate the potential of early arginine or glutamine supplementation in promoting fish growth and enhancing the ability of fish to withstand challenging water temperatures (32 ºC). Zebrafish eggs (*n* = 250 per replicate) were subjected to one of the three experimental conditions, done in triplicate: CTRL) representing the control group without amino acid supplementation, ARG) representing the group with arginine supplementation and, GLN) representing the group with glutamine supplementation. Arginine (A5006, Sigma-Aldrich) and glutamine (G8540, Sigma-Aldrich) were dissolved in Ringer solution for freshwater teleost fish (116 mM NaCl, 2.9 mM KCl, 1.8 mM CaCl_2_, 5 mM Hepes; Young 1933) to achieve a final concentration 50-fold higher than the concentration of the amino acid present in zebrafish eggs (in house determination). Working concentrations were 0.32 mM for arginine and 0.12 mM for glutamine.

**Fig. 1:**
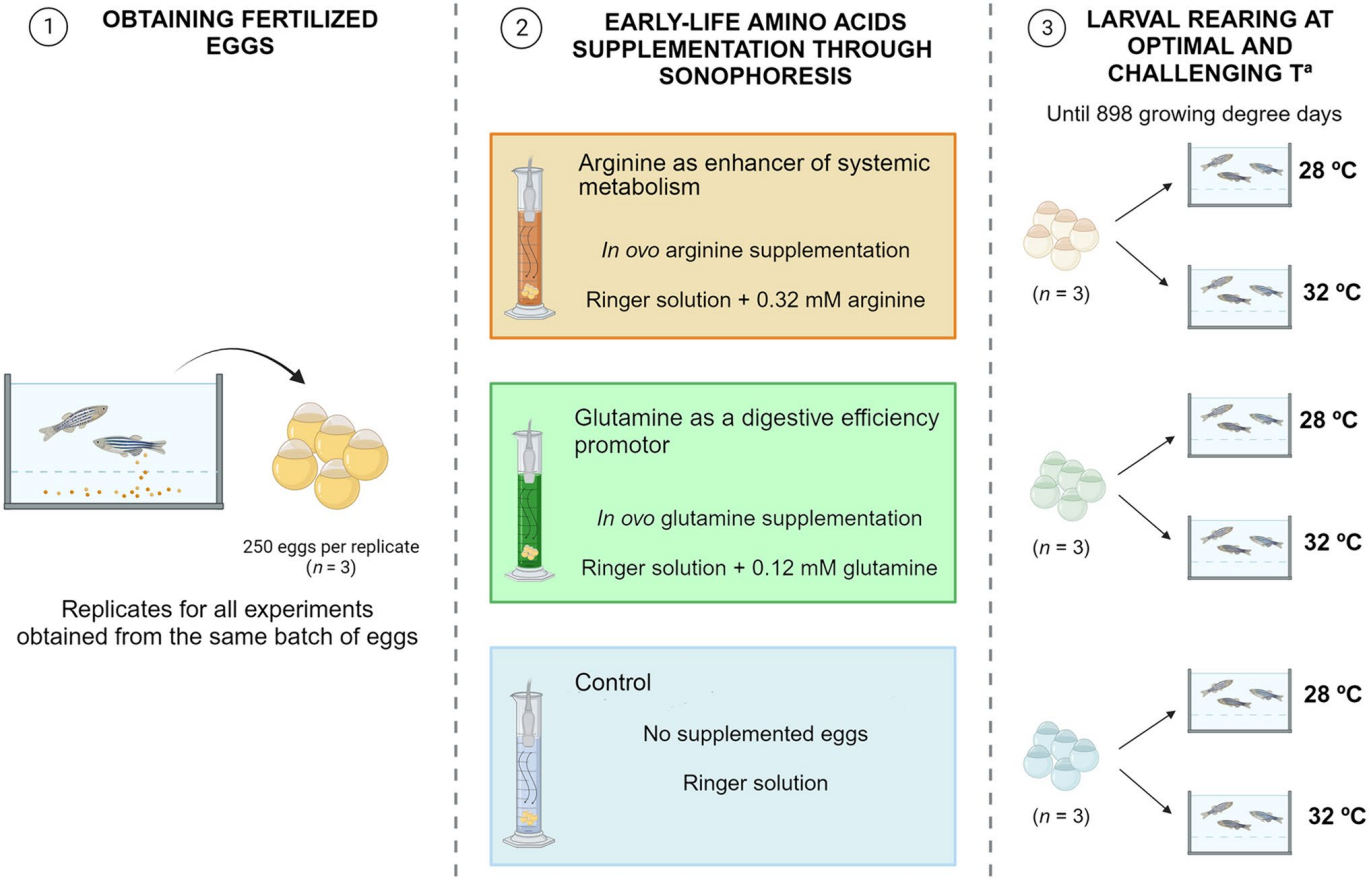
Graphical summary of the experimental design. Created with BioRender.com

Figure 1 provides an overview of the experimental design.

### Early-life amino acids supplementation through sonophoresis

Fertilized eggs were obtained from a breeding population of zebrafish wild-type AB strain (ZFIN ID: ZDB-GENO-960809-7) maintained at the Centre of Marine Sciences of Algarve (CCMAR, Portugal). Sonophoresis technique was used for in ovo supplementation. The sonophoresis prototype system is comprised of a signal generator, a signal amplifier, and an ultrasound immersion transducer, which is submerged in a 250 mL glass measuring cylinder containing the eggs and the solution with the compound to be incorporated (Engrola et al. 2014; Lopes et al. 2016; Navarro-Guillén et al. 2021). The protocol for zebrafish eggs supplementation consisted in two pulses of 100 s duration each (5 s interval between pulses), with a frequency of 1 × 105 Hz, and 100 mV amplitude (Navarro-Guillén et al. 2021).

Initial in-house dose-response trials have established an optimal supplementation dose of 50-fold to attain maximum amino acids’ incorporation. The free amino acid (FAA) profile of zebrafish eggs was employed as a reference for determining the incorporation rate of selected amino acids (unpublished data). For each replicate, 250 eggs were placed in the glass with 150 mL of the respective solution. One hour after sonophoresis 50 eggs from each replicate were sampled to assess the incorporation efficiency of each amino acid (Navarro-Guillén et al. 2021). Eggs were washed twice in distilled water before sampling. Additionally, with the aim of evaluating the effect of sonophoresis technique on the fish egg viability, 50 eggs were submitted to sonophoresis technique without amino acid supplementation, Ringer solution (CTRL SONO), in triplicate (*n =* 3). These eggs were reared at the same stocking density and conditions as group with eggs not subjected to the sonophoresis technique (NO SONO), in triplicate. The hatching rate was evaluated at 3 days post-fertilization (dpf).

### Rearing conditions

After sonophoresis, the remaining 200 eggs from each replicate were divided in two groups of 100 eggs, one group was incubated at 28 ºC and the second group at 32 ºC, at an initial density of 100 eggs L^−1^. Each replicate was placed in an individual experimental unit of 21 × 10.5 × 11 cm size. Larvae were reared until 898 growing degree days (GDD, equivalent to 40 days post-fertilization). The growing degree-days were calculated according to Neuheimer and Taggart (2007). Larvae rearing was done in a flow-through system, at a water temperature of 28 ± 0.8 ºC (28 ºC treatment) or 32 ± 0.3 ºC (32 ºC treatment), a photoperiod of 14 L:10D, dissolved oxygen was kept at 6.7 ± 0.5 mg L^−1^ and pH 7.9 ± 0.2. Larvae were fed *ad libitum* with a commercial inert diet for zebrafish (ZebraFeed, SPAROS Lda., Portugal) from mouth opening (106 GDD).

### Samples collection

At the end of the experimental period (898 GDD) larvae were sampled to assess growth performance, free amino acids (FAA) profile, methylation index, digestive capacity, and intermediary metabolism enzymatic activities. At sampling, larvae were euthanized through rapid cooling (Wallace et al. 2018) and rinsed in distilled water. Fifteen larvae per replicate (*n =* 45 fish per experimental condition) were sampled for dry weight and digestive enzymes activity determination. Three pooled larvae per replicate (*n =* 3 pools per experimental condition) for FAA profile and methylation index, and four pooled larvae per replicate (*n =* 3 pools per experimental condition) were collected for metabolic enzymes activities determination. After collection, all samples were snap-frozen in liquid nitrogen and kept at −80 ºC until analysis. Dry weight measurements were obtained after freeze-drying the samples using a high precision microbalance (± 0.001 mg; MSA36S-000-DH, Sartorius, Germany); previously, larvae were washed twice in distilled water and snap-frozen in liquid nitrogen. Total length was performed using the Leica Application Suite LAS (Leica Microsystems, Wetzlar, Germany) for digital image analysis.

### Biochemical analyses

To evaluate the amino acid incorporation efficiency, the free amino acids (FAA) profile of the zebrafish eggs was analysed 1 h after the procedure. In addition, at the end of the trials (898 GDD), FAA profile of the larvae was assessed to evaluate the modulation of metabolism by early supplementation with amino acids. The relative delta variation (Δ) in amino acid content represents the change in FAA content measured between two distinct groups, specifically before (CTRL) and after the supplementation (ARG or GLN). This variation serves as a quantitative measure, indicating the extent of alteration or enhancement in the FAA content resulting from the supplementation intervention that was calculated using the following formula:

$$\:Relative\:delta\:variation\:\left({\Delta\:}\right)\:=\frac{Sup\:AA-Ctrl\:AA}{Sup\:AA}*100$$

where Sup AA and Ctrl AA are the amino acid concentration (mg AA g larva DW^−1^) in the supplemented (ARG or GLN) and control (CTRL) groups, respectively. Positive delta values signify an increase, while negative values indicate a decrease in amino acid content, providing insights into the impact of supplementation on the overall amino acid profile.

S-adenosylmethionine (SAM) and s-adenosylhomocysteine (SAH) were measured as indicators of methylation index in larvae. Prior to analysis, all samples were freeze-dried. FAA, SAM and SAH analyses were performed after sample homogenization on ice (0.1 M HCl), centrifugation (1,500 g, 4 ºC, 15 min) and deproteinization of the supernatant by centrifugal ultrafiltration (10 kDa cut-off, 2,500 g, 4 ºC, 20 min). Samples were precolumn derivatized with Waters AccQ Fluor Reagent (6-aminoquinolyl-N-hydroxysuccinimidyl carbamate) using the AccQ Tag method (Waters, Milford, MA). SAM and SAH samples were prepared, homogenized and deproteinized with the same protocol as for FAA analysis, but they were not derivatized. All analyses were performed by ultra-high-performance liquid chromatography (UPLC) on a Waters Reversed-Phase Amino Acid Analysis System, using norvaline as an internal standard (Teodósio et al. 2022). Amino acids were identified by retention times of standard mixtures (Waters) and pure standards (Sigma-Aldrich). Instrument control and data acquisition and processing were achieved through Waters Empower software.

### Digestive enzyme activity

The enzymatic extracts were prepared from freeze-dried larvae. Each single larva was manually homogenized in 210 µL of distilled water and then centrifuged (11,000 g, 4 ºC, 10 min) to remove cellular debris. The supernatant extract was used for the analysis of trypsin, chymotrypsin, lipase, amylase, and alkaline phosphatase activities. All samples were kept on ice during the process described above to avoid enzymes denaturation and/or degradation. Enzyme extracts were kept at − 20 ºC until further analysis.

For trypsin and chymotrypsin analysis, the fluorogenic substrates Boc-Gln-Ala-Arg-7-methylcoumarin hydrochloride (B4153, Sigma-Aldrich) and N-Succinyl-Ala-AlaPro-Phe-7-amido-4-metilcoumarin (S9761, Sigma-Aldrich), respectively, were diluted in dimethyl sulfoxide (DMSO) to a final concentration of 20 µM. For analysis, 190 µL of 50 mM Tris + 10 mM CaCl2 buffer (pH 8.5), 15 µL of the extract homogenate and 5 µL of the fluorogenic substrate were added to the microplate. Fluorescence was measured at 355 nm (excitation) and 460 nm (emission).

Lipase activity was assayed using 4-methylumbelliferyl oleate (75164, Sigma-Aldrich). The substrate was dissolved in phosphate buffer pH 7.0 to a final concentration of 0.4 mM (modified from Rotllant et al. 2008), aliquoted and stored at − 20 ºC. For analysis, the larvae homogenate (15 µL) was added to the microplate and mixed with 250 µL of 0.4 mM substrate for the analysis. Fluorescence was measured at 355 nm (excitation) and 460 nm (emission).

EnzChek™ Ultra Amylase Assay Kit (E33651, Invitrogen™) was used for amylase analysis. This kit contains a starch derivate labelled with a fluorophore dye as substrate. This substrate was diluted in 3-(N-morpholino) propanesulfonic acid (MOPS; pH 6.9) and substrate solvent (sodium acetate; pH 4.0), to a final concentration of 200 µg mL^−1^. For analysis, 50 µL of the substrate solution and 15 µL of the larvae extract were added to the microplate. Fluorescence was measured at 485 nm (excitation) and 538 nm (emission).

For alkaline phosphatase analysis the substrate 4-methylumbelliferyl phosphate disodium salt (4-MUP, M8168, Sigma-Aldrich) was diluted in borate buffer (pH 8.5) to a final concentration of 1 mM. In the microplate, 100 uL of substrate and 15 uL of extract were mixed. Fluorescence was measured at 360 nm (excitation) and 440 nm (emission).

For all enzymes the liberation of the fluorophore was kinetically followed during 5 min at a standard temperature of 37 ºC in analytical triplicates, and activities were expressed as RFU (Relative Fluorescence Units) per mg of larva (dry weight).

### Metabolic enzyme activity

The enzymatic activities were analysed in lyophilized larvae (*n* = 3 pool per experimental condition). Pooled larvae were weighed and then homogenized by mechanical disruption in ice-cold buffer (50 mM imidazole, 1 mM 2-mercaptoethanol, 50 mM NaF, 4 mM EDTA, 0.5 mM phenylmethylsulfonyl fluoride (PMSF) and 250 mM sucrose; pH 7.5). The homogenate was centrifuged (3,220 g, 4 °C, 30 min), and the supernatant stored at − 80 °C for further analysis. The assays of GP (glycogen phosphorylase, EC 2.4.1.1), PK (pyruvate kinase, EC 2.7.1.40), HK (hexokinase, EC 2.7.1.1), GPDH (glycerol-3-phosphate dehydrogenase, EC 1.1.1.8), HADH (3-hydroxyacyl-CoA dehydrogenase, EC 1.1.1.35), ALT (alanine aminotransferase, EC 2.6.1.2), AST (aspartate aminotransferase, EC 2.6.1.1), GLDH (glutamate dehydrogenase, EC 1.4.1.2), FBP (fructose 1,6-bisphosphatase, EC 3.1.3.11) and LDH (lactate dehydrogenase, EC 1.1.1.27) were performed as previously described Laiz-Carrión et al. (2003); Sangiao-Alvarellos et al. (2005, 2006); Polakof et al. (2007); Vargas-Chacoff et al. (2016) and Jerez-Cepa et al. (2020, 2021). These protocols were similarly described and validated for zebrafish (Faught and Vijayan 2019a, 2019b). Enzyme activities were determined in analytic triplicates using a PowerWave™ 340 microplate spectrophotometer (Bio-Tek Instruments, Winooski, VT, USA) using KCjunior™ data analysis software for Microsoft^®^. Reaction rates of enzymes were determined by changes in absorbance at 340 nm from the redox reaction of NAD(P) + to NAD(P)H or vice versa. The total activity, expressed as standard units (U; µmol min-1), was normalized by dry weight and the protein concentration. Total protein concentration was determined in duplicate with a BCA Protein Assay Kit (Pierce™, Thermo Fisher Scientific, USA, #23225) using BSA as a standard.

### Statistics

Data of survival, individual dry weight, FAA, SAM, SAH and activity levels of digestive and metabolic enzymes are shown as means ± standard deviation (SD). Percentage data (hatching rate and survival) were arcsine square root-transformed prior to analysis. Hatching rate between NO SONO and CTRL SONO groups was compared by means of unpaired two-tailed Student’s *t*-test. All data were checked for normality and homogeneity of variance using Kolmogorov-Smirnov and Levene’s tests, respectively. To evaluate the impact of amino acid supplementation within each rearing temperature on survival, growth performance, methylation index, and enzymatic activity levels, two-way ANOVA was employed. Differences among means were detected by the Tukey’s test. All other variables (paired data) were compared by two-tailed Student’s *t*-test. All analyses were performed with SPSS 26 software (IBM, New York, USA). Statistical significance was accepted at *p* < 0.05.

## Results

The sonophoresis technique did not have significant negative effect on zebrafish larvae hatching rate; as the average hatching rate in eggs not submitted to sonophoresis (NO SONO group) was 95 ± 55%, and the hatching rate in eggs submitted to sonophoresis (CTRL SONO) was 85 ± 8% (*p* = 0.112).

Neither rearing temperature nor amino acids supplementation affected the survival of zebrafish larvae (Table 1, *p* > 0.05). Overall, survival after 898 GDD was 51.8 ± 23.1%. Growth performance in terms of weight and length, was significantly higher for larvae reared at optimal temperature (28 ºC, *p* < 0.001), with an average final dry weight of 6.5 ± 2.7 mg larva^−1^ and furcal length of 13.9 ± 1.7 mm (Table 1). Amino acids supplementation had no impact on growth indicators, however, although supplementation factor was not significant (*p* = 0.084) at end of experimental period ARG larvae tended to be heavier than CTRL larvae at higher temperature (32 ºC) (Table 1). Statistical results for biometric data are shown in Supplementary File 1.

**Table 1:** Zebrafish larvae survival (%) and growth performance (individual dry weight, DW [mg larva^−1^] and furcal length, FL [mm]) at the end of the trials (898 GDD)

	CTRL			ARG			GLN	
	28 ºC	32 ºC		28 ºC	32 ºC		28 ºC	32 ºC
Survival (%)	52.0 ± 41.6	46.3 ± 12.7		54.0 ± 31.2	47.3 ± 14.8		61.3 ± 30.2	54.0 ± 9.9
DW (mg larva^−1^)	5.7 ± 2.4	1.6 ± 0.7		6.5 ± 2.7	2.9 ± 0.9		7.4 ± 3.1	2.0 ± 0.7
FL (mm)	13.4 ± 1.6	11.0 ± 1.2		13.7 ± 1.7	10.3 ± 0.5		14.7 ± 1.7	10.4 ± 1.3

Results are shown as mean ± SD

### Biochemical analyses

#### Amino acid incorporation efficiency

The free amino acid (FAA) profile of zebrafish eggs (1 h after sonophoresis) confirmed the effectiveness of sonophoresis for arginine (ARG) and glutamine (GLN) supplementation into zebrafish eggs. Eggs from ARG group presented higher levels of arginine compared to CTRL (*p* = 0.002), with 16.01 ± 0.7 and 7.89 ± 0.13 mg arginine g egg DW^−1^, respectively, which translates into an incorporation rate of 102.77% in the ARG group. Additionally, eggs from GLN group presented higher levels of glutamine compared to CTRL eggs (11.50 ± 0.67 and 3.46 ± 0.05 mg glutamine g egg DW^−1^, respectively. *p* < 0.001). Incorporation rate in glutamine-supplemented eggs was 232.35%. Free amino acids (FAA) profile of zebrafish eggs (1 h after sonophoresis) is presented in Table 2.

**Table 2:** Zebrafish eggs free amino acids profile (mg AA g DW egg^−1^) 1 h after supplementation with sonophoresis for control (CTRL), arginine (ARG) and glutamine (GLN) experimental conditions

	1 h after sonophoresis eggs		
	CTRL	**ARG**	**GLN**
Arginine	7.89 ± 0.13	16.01 ± 0.71^*^	7.78 ± 0.37
Glutamine	3.46 ± 0.05	3.74 ± 0.22	11.50 ± 0.67^*^

Results are shown as mean ± SD. Asterisk (*) means statistical differences between CTRL and experimental groups (ARG and GLN)

**Fig. 2:**
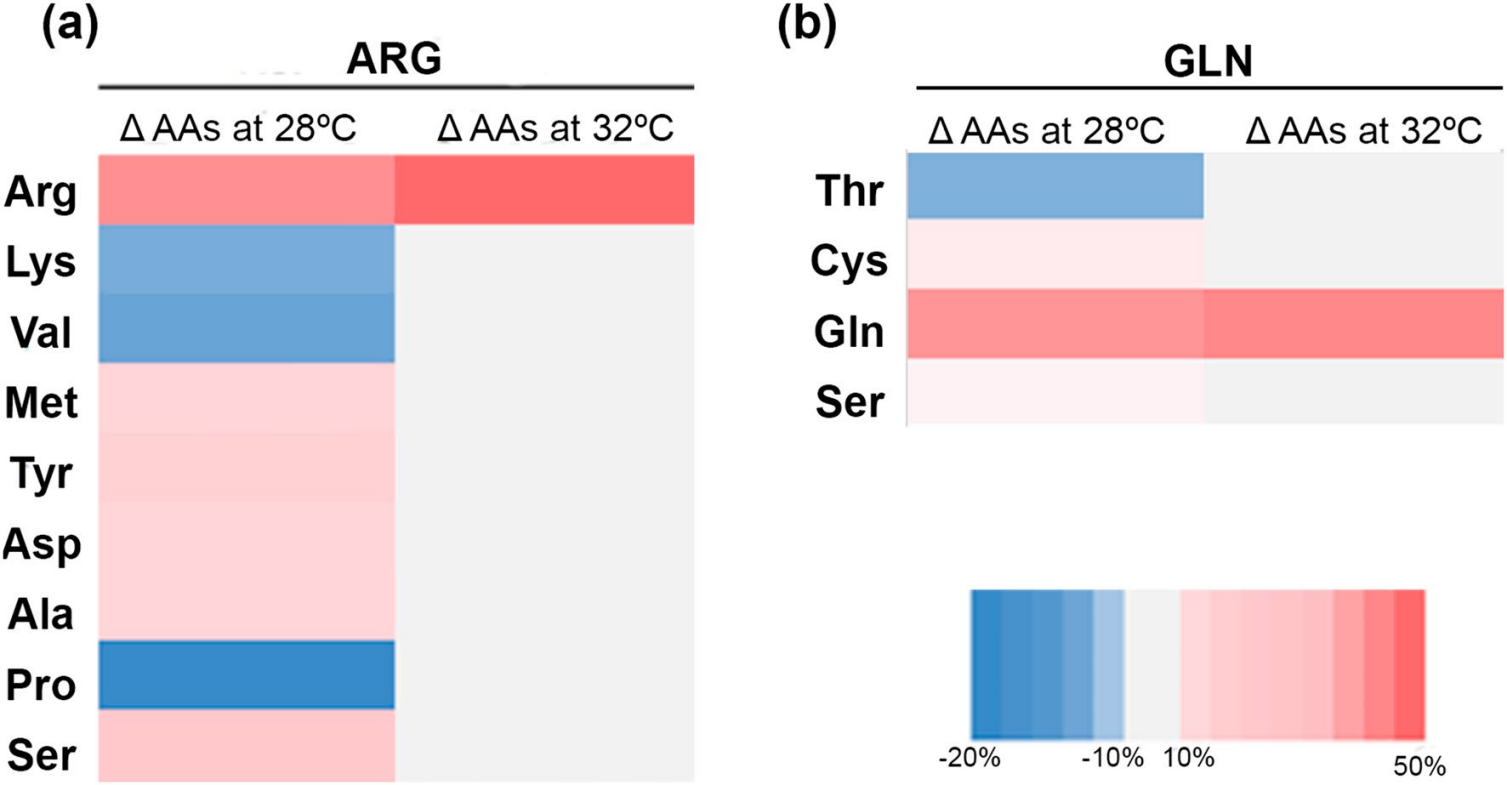
Relative delta variation in the larval free amino acid profile of larvae at the end of the experimental period (898 GDD) supplemented with arginine **a** or glutamine **b** expressed as % of the supplemented group (*n* = 3 pools of 3-pooled larvae per experimental condition. The plot represents the most variable amino acids ( > ± 10% of relative delta variation)

Variations in the amino acid profile of arginine-supplemented larvae compared to control larvae were more evident at 28 ºC (Fig. 2a). At the end of the experiment, ARG-larvae reared at 28 ºC showed a relative delta variation in arginine content of 36.9%. On the other hand, the indispensable amino acids lysine and valine decreased in the supplemented larvae, with relative delta variations of −12.9 and −14.3%, respectively. The same was observed for the dispensable amino acid proline, with a relative delta variation of −18.4%. In fish reared at 32 ºC variations in FAAs profile were only recorded for arginine, which presented a relative delta variation of 48.7%. For glutamine-supplemented larvae, variations in the amino acid profile compared to control larvae only revealed four amino acids with high degree of delta variation ( > ± 10%) between experimental groups at 28 ºC (Fig. 2b). At the end of the experiment, GLN-larvae reared at 28 ºC showed a relative delta variation in glutamine content of 36.4%. The indispensable amino acid threonine decreased in the supplemented larvae, with a relative delta variation of −10.1%. On the other hand, the indispensable amino acids cysteine and serine showed a relative delta variation of 12.5 and 10.1%, respectively. In fish reared at 32 ºC variations in FAAs profile were only recorded for glutamine, which presented a relative delta variation of 40.0%.

#### Methylation index

**Fig. 3:**
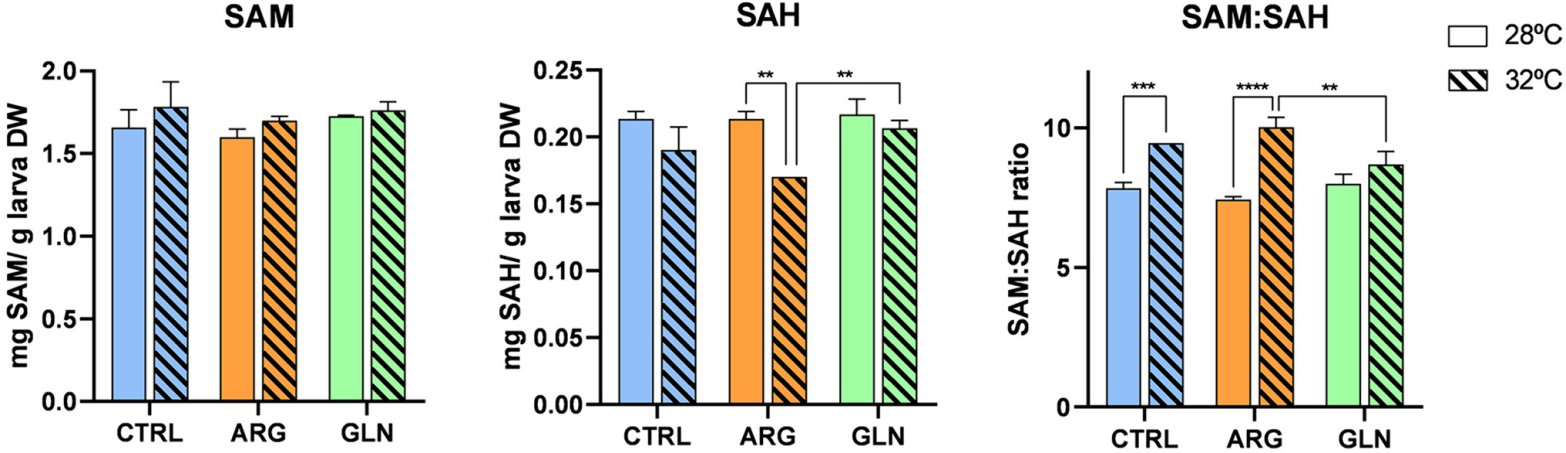
Zebrafish larvae methylation index at the end of the experimental period (898 GDD). Concentration of s-adenosylmethionine (SAM), s-adenosylhomocysteine (SAH) and SAM: SAH as indicator of methylation index (mg g larva DW^−1^) in zebrafish larvae from control (CTRL, blue bars), arginine-supplemented (ARG, orange bars) and glutamine-supplemented (GLN, green bars) groups and reared at 28 ºC (plain bars) or 32 ºC (striped bars). Asterisks indicate statistical differences between experimental groups (** *p* < 0.01; *** *p* < 0.001; **** *p* < 0.0001)

Levels of SAM, SAH and SAM: SAH ratio are shown in Fig. 3. Overall, rearing temperature showed a significant effect in the three variables (*p* < 0.05). SAH content was higher in fish reared at 28 ºC while SAM, and consequently SAM: SAH ratio, were higher in fish at 32 ºC. In addition, SAH concentration was significantly affected by the supplementation condition (*p* = 0.016) and the interaction between temperature and treatment (*p* = 0.016). SAH levels in ARG-larvae reared at 32 ºC decreased, being statistically lower than larvae from the same experimental condition reared at 28 ºC and also lower than GLN-larvae at 32 ºC. Likely, SAM: SAH ratio was also affected by the interaction of temperature and experimental condition (*p* < 0.001). An increase in water temperature led to an increase in SAM: SAH ratio in CTRL and ARG-larvae compared to their homologous at 28 ºC. This effect of temperature on the methylation potential was not observed for GLN-larvae, maintaining similar values at both temperatures. Two-way ANOVA results for methylation index are shown in Supplementary File 1.

### Digestive enzymes activity levels

**Fig. 4:**
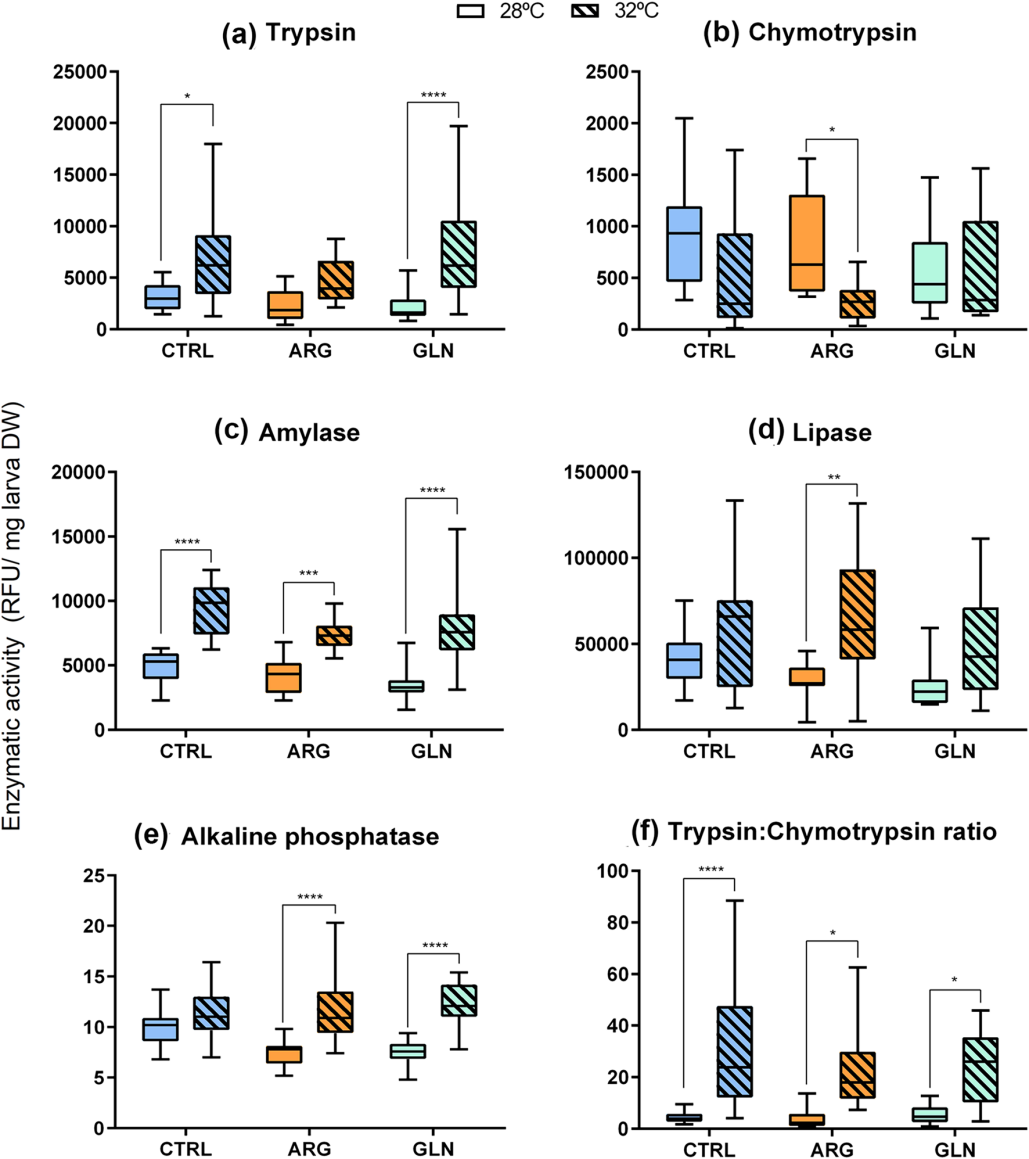
Zebrafish larvae digestive enzymes activity levels. Trypsin (**a**), chymotrypsin (**b**), amylase (**c**), lipase (**d**) and alkaline phosphatase (**e**) activity levels and trypsin: chymotrypsin ratio (**f**) in zebrafish larvae (898 GDD) from control (CTRL, blue bars), arginine (ARG, orange bars) and glutamine (GLN, green bars) groups reared at 28 ºC (plain bars) or 32 ºC (striped bars; *n* = 15). Activity values are expressed as RFU per mg larva^−1^. The horizontal line within the box represents the median value. Asterisks indicate statistical differences between experimental groups (* *p* < 0.05; ** *p* < 0.01; *** *p* < 0.001; **** *p* < 0.0001)

Rearing temperature affected all digestive enzymes activity levels (Fig. 4, *p* < 0.05). Overall, larvae reared at 32 ºC presented higher activity levels for all enzymes apart from chymotrypsin (Fig. 4b), which tended to decrease. However, this decrease was only statistically significant for the ARG-group. For lipase activity (Fig. 4d), the increase in activity with temperature was only significant for ARG-larvae, while for trypsin activity (Fig. 4a) it was observed the opposite pattern, being only significant for CTRL and GLN-groups. For alkaline phosphatase activity (Fig. 4e), differences between temperatures were only found in the supplemented groups (ARG and GLN). In addition, for alkaline phosphatase a significant interaction between water temperature and supplemental condition was found (*p* = 0.008). Two-way ANOVA results for digestive enzymes activity levels are shown in Supplementary File 1.

### Metabolic enzymes activity

Intermediary metabolism was affected by water temperature. Overall, the activity of all metabolic enzymes was significantly higher in larvae reared at 28 ºC than in larvae reared at 32 ºC (Table 3, *p* < 0.05). Supplementary condition was only statistically significant for PK, ALT and GLDH (*p* = 0.041, 0.007 and 0.001, respectively). By contrast, a statistical interaction between both factors was observed for most of the enzymes, except for FBP, GPDH, ALT and AST (*p* = 0.823, 0.239, 0.119 and 0.229, respectively). Two-way ANOVA results for metabolic enzymes are shown in Supplementary File 1.

**Table 3:** Intermediate metabolism of zebrafish larvae

	CTRL		ARG		GLN	
	28º C	32 ºC	28 ºC	32 ºC	28 ºC	32 ºC
Carbohydrates metabolism						
GP	0.17 ± 0.07^a^	0.01 ± 0.01^c^	0.14 ± 0.08^ab^	0.13 ± 0.03^abc^	0.18 ± 0.09^a^	0.04 ± 0.02^bc^
HK	0.09 ± 0.03^b^	0.05 ± 0.02^b^	0.21 ± 0.12^a^	0.04 ± 0.02 ^b^	0.04 ± 0.02^b^	0.08 ± 0.04 ^b^
PK	2.31 ± 0.59^bc^	0.93 ± 0.52^c^	4.33 ± 1.22^a^	0.92 ± 0.14^c^	2.98 ± 1.04^ab^	0.88 ± 0.33^c^
LDH	4.67 ± 0.43^a^	2.66 ± 0.78^b^	6.05 ± 0.83^a^	2.22 ± 0.26^b^	5.93 ± 1.33^a^	2.11 ± 0.52^b^
FBP	0.34 ± 0.20	0.06 ± 0.03	0.29 ± 0.20	0.10 ± 0.04	0.31 ± 0.26	0.04 ± 0.03
Lipids metabolism						
HADH	0.15 ± 0.04^bc^	0.10 ± 0.06^bc^	0.20 ± 0.05^ab^	0.10 ± 0.05^bc^	0.30 ± 0.09^a^	0.06 ± 0.02^c^
GPDH	0.14 ± 0.08	0.15 ± 0.06	0.26 ± 0.10	0.06 ± 0.05	0.24 ± 0.27	0.08 ± 0.03
Amino acids metabolism						
ALT	0.46 ± 0.20	0.34 ± 0.14	0.81 ± 0.13	0.42 ± 0.08	0.59 ± 0.10	0.25 ± 0.10
AST	3.23 ± 0.74	1.45 ± 0.25	4.17 ± 1.15	1.23 ± 0.29	3.85 ± 1.05	0.95 ± 0.30
GLDH	0.65 ± 0.03^b^	0.32 ± 0.09^c^	0.99 ± 0.08^a^	0.37 ± 0.10^c^	0.70 ± 0.14^b^	0.30 ± 0.08^c^

Results are shown as mean ± SD (*n* = 4). Data were normalized by dry weight of larvae and the protein concentration of homogenate. Different lowercase (a, b,c) letters mean statistical differences between experimental groups (Tukey’s test, *p* < 0.05). Metabolic enzymes activities (U µmol min^−1^) in zebrafish larvae (898 GDD) from control (CTRL), arginine (ARG) ang glutamine (GLN) groups reared at 28 ºC and 32 ºC. *GP* glycogen phosphorylase, *HK* hexokinase, *PK* pyruvate kinase, *LDH* lactate dehydrogenase, *FBP* fructose 1,6-bisphosphatase, *HADH* 3-hydroxyacyl-CoA dehydrogenase, *GPDH* glycerol-3-phosphate dehydrogenase, *ALT* alanine aminotransferase, *AST* aspartate aminotransferase, *GLDH* glutamate dehydrogenase

When focusing on carbohydrates metabolism, most of the enzymes were affected by the interaction of water temperature and egg amino acid supplementation, with the only exception of FBP. HK and PK activity levels in 28-ARG larvae were higher when compared to larvae from the remain treatments independently of the water temperature, while although no significant differences were found between groups at 28 ºC for GP activity, 32-ARG larvae was the only group reared at 32 ºC that maintained similar activity levels to its homologous at 28 ºC.

Regarding the metabolism of lipids, the activity of HADH was significant affected by the interaction of temperature and amino acid supplementation (*p* = 0.001). Enzymatic activity was enhanced in supplemented GLN-larvae in comparison to CTRL group at 28 ºC. By contrast, while CTRL and ARG groups kept similar activity values at both rearing temperatures, in GLN-larvae activity statistically decreased with temperature.

Concerning the metabolism of amino acids, ALT activity was significantly affected by amino acid supplementation (*p* = 0.007), which activity was higher in ARG larvae. On the other hand, a significant interaction between water temperature and supplementation was found for GLDH (*p* = 0.015), with the highest activity levels recorded in 28-ARG larvae.

## Discussion

This study assesses in ovo amino acid supplementation as a potential strategy to foster climate-resilient fish. With further studies it could be adapted to target both freshwater and marine farmed fish species. Sonophoresis proved to be a harmless technique, as it exhibited no impact on egg hatching rate. Survival rate at the end of the experiment was not affected by rearing temperature or amino acids supplementation, with an average survival of 51% at 898 GDD. Survival results are consistent to those reported by Navarro-Guillén et al. (2021) for zebrafish larvae at 22 dpf after in ovo amino acid supplementation. The embryonic development of zebrafish is successful between 22 ºC and 32 ºC, with high survival proportional to hatching (Schnurr et al. 2014), and with non-significant differences in larvae survival between 28 °C and 32 °C (Zhang et al. 2018). However, 32 ºC is considered a sublethal temperature for zebrafish, being the extreme of the temperature range that permits growth due to over-activation of metabolism, since optimal temperature for rearing is 28 ± 0.5 °C (Westerfield 2000; López-Olmeda and Sánchez-Vázquez 2011; Scott and Johnston 2012; Pype et al. 2015). The results from the present study are in agreement since survival was unaffected however growth was depressed at 32 ºC, suggesting that larvae were allocating energy for metabolic purposes to cope with a challenging temperature rather than growth.

The viability of sonophoresis technique for in ovo nutritional modulation in fish was supported by the incorporation efficiency results, as well as survival and hatching rates. Arginine and glutamine levels were higher in ARG and GLN eggs, respectively. Although the solutions used for sonophoresis were 50-fold more concentrated than arginine and glutamine levels in zebrafish eggs, the incorporation rates were 102.77 and 232.35% for ARG and GLN, respectively. These results are aligned with those described in Lopes (2016) for methionine supplementation in gilthead seabream eggs, and Navarro-Guillén et al. (2021) for zebrafish eggs, suggesting that the incorporation efficiency may differ between species and type of amino acid. It is interesting to remark that larvae FAA profile after 898 GDD maintained the modulation induced in eggs for each experimental condition, with higher arginine and glutamine content in ARG and GLN experimental groups, respectively. Previous studies have assessed in ovo AA supplementation, many of them in broilers, but few measured the lasting effects on FAA profile after hatching. Interestingly, in agreement with our present study, Yu et al. (2018) recorded higher arginine levels in the muscle of 21-day post-hatch broilers after in ovo supplementation of arginine. Moreover, we also found the same pattern in our previous study Navarro-Guillén et al. (2021). Thus, it seems that the supplemented AAs are not compensated or metabolized along the embryonic/early development, suggesting an imprint on the regulation of AA metabolism. However, further studies including techniques to trace the fate of specific amino acids are needed to confirm this hypothesis (e.g. isotopic tracer studies).

The effect of high-water temperature on growth performance resulted in both decreased weight and length in larvae reared at 32 ºC in both experiments. Previous studies suggested that an exposure to high temperatures during long periods may have a significant energetic cost in zebrafish larvae decreasing growth rate and this effect persists into later life (Schnurr et al. 2014). Thus, larvae from eggs incubated at 32 °C tended to perform smaller at 8 and 12 weeks after hatching than larvae from eggs incubated at 22 or 27 ºC, even though larvae were transferred to 27 °C at hatching (Schnurr et al. 2014). A decreased growth rate in larvae exposed to elevated temperatures has been reported for other species such the Atlantic herring (*Clupea harengus*) (Sswat et al. 2018). By contrast, a faster larval growth at warmer temperatures has been reported for the European seabass (*Dicentrarchus labrax*), golden pompano (*Trachinotus ovatus*) and Senegalese sole (Pimentel et al. 2014; Cominassi et al. 2019; Han et al. 2020). In gilthead seabream, larvae reared at 18 ºC showed greater growth performance in terms of weight, length, and survival than larvae at 16 and 20 ºC (Azab et al. 2015). However, in these studies the warmer temperature led to lower hatching and survival rates, suggesting a trade-off between survival and fast growth. According to these contradictory results, it can be assumed that the effect of temperature on fish growth and development at early life stages is species-specific. A possible reason for the negative effect of high temperature on growth could be the higher energy demand derived from increased metabolic rates (Rubalcaba et al. 2020). The composition of the free amino acid (FAA) pool exhibited several changes at the optimal temperature of 28 ºC in both experiments. However, the positive variations observed in the supplemented groups at 28 ºC were no longer apparent at 32 ºC, except for the target amino acids arginine and glutamine. The decrease in the FAA pool at 32 ºC, tightly regulated by the fish (Conceição et al. 2012), indicates that larvae were likely utilizing amino acids for metabolic purposes, such as activating pathways to cope with challenging environmental conditions. The tendency to higher weight of ARG-larvae at 32 ºC underscores the positive impact that in ovo arginine supplementation may have on fish larvae metabolism, enabling the animals to better cope with challenging conditions.

The delta variations in the free amino acids (FAAs) profile at the optimal temperature of 28 ºC indicate distinctions in amino acid metabolism based on the nature of the supplemented amino acid, specifically, whether it is indispensable or dispensable. Arginine, as a multifunctional amino acid, participates in various metabolic pathways. These pathways are interconnected, and changes in one pathway can influence others (Mommsen 2001). Several arginine metabolites are intricately linked to growth, and muscle growth. The early intervention with arginine, the lasting effect observed in the larvae, and the amino acid multifunctionality could explain the tendency to a higher growth observed in the fish. This may also be linked with the observed reduction of lysine and valine, two amino acids directly involved in muscle accretion (Valente et al. 2013). Glutamine holds a pivotal role in maintaining gut balance as it serves as the preferred substrate for rapidly dividing cells, as highlighted by Bertrand et al. (2013). As the gut mucosa undergoes constant renewal, fish must continuously produce mucins for maintaining the mucosal barrier. Mucin is a glycoprotein that includes threonine (Bansil and Turner 2018). A reduction in threonine in GLN-larvae could suggest a positive effect of an early intervention with glutamine in gut homeostasis. However, further studies assessing the specific metabolism of the supplemented amino acids are needed to confirm this hypothesis.

Adaptation to changing environments through phenotypic plasticity may be mediated by modulation of gene expression through DNA methylation (Beemelmanns et al. 2021). The transfer of a methyl group from the universal methyl donor SAM to the 5-position of cytosine residues in DNA is mediated by DNA methyltransferases (DNMTs). The methylation index, defined as the ratio of SAM and SAH (formed by the demethylation of SAM), is one of the best ways to evaluate methylation status in organisms, which is dependent on an adequate SAM supply and SAH removal (Kadayifci et al. 2018). However, a decrease in SAM: SAH ratio has been described as predictive of increased methylation capacity especially when associated with a decrease in SAH (Caudill et al. 2001). In the present study, although water temperature increased SAM: SAH ratio in CTRL and ARG-larvae, SAH levels only decreased in the ARG group, suggesting greater methylation capacity in zebrafish larvae supplemented with arginine. These results agree with those previously described for other fish species under thermal stress. In the Atlantic salmon (*Salmo salar*), high water temperature induced dynamic DNA methylation changes and, consequently, affected the regulation of the expression of genes involved in heat shock response, mRNA stability, cellular oxidative stress response and apoptosis (Beemelmanns et al. 2021). In European seabass larvae, elevated temperatures promoted a developmental stage-dependent effect on global DNA methylation and overexpression of *dnmt1*, *dnmt3* and *myogenin* (*myog*) (Anastasiadi et al. 2017). By contrast, in Senegalese sole larvae, a lower rearing temperature upregulated the expression of *dnmt1* and *dnmt3b* promoting *myog* methylation, which was translated into a downregulation of *myog* transcription (Campos et al. 2013c). All together, these results illustrate the species-specificity impact of environmental factors on fish epigenetic mechanisms.

Efficient digestion of nutrients depends on the availability and activity of digestive enzymes, as well as on the gut transit time. Biochemical reactions, and therefore enzymatic rates, vary with temperature and tend to increase with increasing temperatures (Volkoff and Rønnestad 2020). In Atlantic salmon, trypsin activity levels were higher during Spring compared to Winter (Einarsson et al. 1997). Similarly, in the cunner (*Tautogolabrus adspersus*) and greater amberjack (*Seriola dumerili*), low temperatures reduced intestinal digestive enzymes activities (Hayes and Volkoff 2014; Navarro-Guillén et al. 2022). By contrast, in yellowtail kingfish (*Seriola lalandi*) protease activity in the intestine was higher in winter, probably as a compensatory strategy to slower gut motility at lower water temperatures (Miegel et al. 2010). In the present study, water temperature significantly affected the digestion process, since most enzyme activity levels tended to be higher in fish reared at 32 ºC, except for chymotrypsin. However, this global higher digestive capacity at 32 ºC did not turn into an improved growth performance. At higher temperatures, higher metabolic rates imply higher energy demand, and as result, larval performance could be compromised (Pankhurst 2011). Therefore, the higher activity levels observed in 32 ºC-larvae could be a compensatory mechanism to sustain the body homeostasis derived from a higher energy expenditure together with a shorter residence time of the feed in the gut, driven by a faster transit time (Yúfera et al. 2019; Navarro-Guillén et al. 2023). Positive effects of dietary glutamine on digestive function, through the enhancement of digestive enzyme activity, have been described in several animal species (Yan and Qiu-Zhou 2006; Liu et al. 2015; Wu et al. 2021, 2022), however, in the present study, there was no positive correlation between early intervention with glutamine supplementation and a later activity of digestive enzymes. Further studies assessing also intestinal structure may help understanding how early interventions with glutamine could modulate fish digestive integrity and functionality.

Both acute and chronic exposure to high temperatures is associated with changes in fish intermediary metabolism. The magnitude of the metabolic responses depends on the temperature progression rate towards the upper and lower limits and duration of exposure (Islam et al. 2021). Temperature enhancement is suggested to increase metabolic demand and thus whole-animal metabolic rates (Volkoff and Rønnestad 2020). However, in our study, the metabolic enzyme activities were systematically lower in fish reared at 32 ºC compared to those at 28 ºC, probably due to an excessive consumption of energy substrates that led to a lack or incomplete metabolic compensation for temperature increase. As consequence, the higher demand of energy and the metabolic stress induced resulted in a lower growth performance at 32 ºC (Rubalcaba et al. 2020). Moreover, a combined effect of in ovo supplementation with amino acids and water temperature differentially modulated the metabolic profile of larvae after 898 GDD. Thus, while glutamine only stimulated lipid catabolism at 28 ºC, reflected in increased HADH activity, arginine supplementation enhanced carbohydrates and amino acids catabolism, since HK, PK and GLDH activities were stimulated compared to CTRL group. Arginine has been described as modulator of fish carbohydrate metabolism, with the potential to stimulate glucose uptake by its insulinotropic effect (Andersen et al. 2014, 2016; Wang et al. 2021). In blunt snout bream (*Megalobrama amblycephala*) juveniles, dietary arginine elevated plasma glucose, as well as upregulated GP and PK expression levels, suggesting the promotion of glucose catabolism by arginine (Liang et al. 2017). The results from the present study suggest an enhanced carbohydrate metabolism through HK and PK activity levels induced by arginine supplementation at optimal temperature, 28 ºC. Moreover, at 32 ºC, GP activity in ARG-larvae tended to be higher in comparison to CTRL and GLN-larvae, which can be associated with the same effect. This improved use of energy reserves under challenging conditions might have supported the tendency towards improved growth performance registered in ARG-larvae. Regarding amino acids metabolism, the increased activity of GLDH enzymes in ARG-larvae at 28 ºC can be associated with a stimulation of gluconeogenesis pathways in liver through amino acids catabolism (Faught et al. 2016).

In conclusion, the present work gives insights on how an early nutritional intervention can modulate metabolic responses and alter phenotypic plasticity in fish larvae. Results clearly indicated 28 °C is an optimal water temperature for growth of zebrafish larvae. Nevertheless, results suggest that in ovo arginine supplementation may promote a better metabolic adaptation to cope with higher temperatures. Overall, the presents study elucidate how specific nutrients may change animal response to environmental stressors. Further studies may help understanding how metabolism changes in fish under challenging conditions.

## Electronic supplementary material

Below is the link to the electronic supplementary material.

Supplementary Material 1

## Data Availability

The raw data required to reproduce the above findings are available to download from Zenodo Repository, DOI: 10.5281/zenodo.8380091.
