# Supplementary material for: Effects of early-life amino acids supplementation on fish responses to a thermal challenge
Source: J Comp Physiol B. 2024 Sep 13;194(6):827–42. doi: 10.1007/s00360-024-01581-1 (PMC11511724; doi:10.1007/s00360-024-01581-1)
Supplement: Supplementary file 1 — Supplementary Material 1 [file 360_2024_1581_MOESM1_ESM.docx]

**Supplemental File 1. Two-way ANOVA results**

Biometric data

|  | ***df*** | ***F*** | ***p*** |
| --- | --- | --- | --- |
| *Survival (%)* | | | |
| Temperature | 1 | 0.496 | 0.495 |
| Supplementation | 2 | 0.074 | 0.929 |
| Temp * Suppl | 2 | 0.018 | 0.982 |
| *DW (mg larva^-1^)* | | | |
| Temperature | 1 | 97.743 | <0.001 |
| Supplementation | 2 | 2.554 | 0.084 |
| Temp * Suppl | 2 | 1.428 | 0.246 |
| *FL (mm)* | | | |
| Temperature | 1 | 108.593 | <0.001 |
| Supplementation | 2 | 0.826 | 0.442 |
| Temp * Suppl | 2 | 2.651 | 0.078 |

Methylation index

|  | ***df*** | ***F*** | ***p*** |
| --- | --- | --- | --- |
| *SAM* | | | |
| Temperature | 1 | 5.142 | 0.043 |
| Supplementation | 2 | 2.190 | 0.155 |
| Temp * Suppl | 2 | 0.563 | 0.584 |
| *SAH* | | | |
| Temperature | 1 | 46.730 | <0.001 |
| Supplementation | 2 | 5.998 | 0.016 |
| Temp * Suppl | 2 | 5.908 | 0.016 |
| *SAM:SAH* | | | |
| Temperature | 1 | 141.076 | <0.001 |
| Supplementation | 2 | 2.853 | 0.097 |
| Temp * Suppl | 2 | 16.153 | <0.001 |

Digestive enzymes activity levels

|  | ***df*** | ***F*** | ***p*** |
| --- | --- | --- | --- |
| *Trypsin* | | | |
| Temperature | 1 | 36.779 | <0.001 |
| Supplementation | 2 | 2.764 | 0.069 |
| Temp * Suppl | 2 | 2.086 | 0.131 |
| *Chymotrypsin* | | | |
| Temperature | 1 | 7.775 | 0.007 |
| Supplementation | 2 | 1.052 | 0.355 |
| Temp * Suppl | 2 | 2.015 | 0.141 |
| *Amylase* | | | |
| Temperature | 1 | 90.869 | <0.001 |
| Supplementation | 2 | 3.405 | 0.038 |
| Temp * Suppl | 2 | 1.301 | 0.278 |
| *Lipase* | | | |
| Temperature | 1 | 22.611 | <0.001 |
| Supplementation | 2 | 1.841 | 0.165 |
| Temp * Suppl | 2 | 0.547 | 0.581 |
| *Alkaline phosphatase* | | | |
| Temperature | 1 | 55.816 | <0.001 |
| Supplementation | 2 | 0.935 | 0.397 |
| Temp * Suppl | 2 | 5.103 | 0.008 |
| *Trypsin:Chymotrypsin ratio* | | | |
| Temperature | 1 | 42.957 | <0.001 |
| Supplementation | 2 | 0.908 | 0.408 |
| Temp * Suppl | 2 | 0.763 | 0.471 |

Metabolic enzymes activity

|  | ***df*** | ***F*** | ***p*** |
| --- | --- | --- | --- |
| ***Carbohydrates metabolism*** | | | |
| *GP* | | | |
| Temperature | 1 | 18.480 | <0.001 |
| Supplementation | 2 | 1.173 | 0.332 |
| Temp * Suppl | 2 | 3.827 | 0.041 |
| *HK* | | | |
| Temperature | 1 | 6.387 | 0.021 |
| Supplementation | 2 | 3.249 | 0.062 |
| Temp * Suppl | 2 | 7.448 | 0.004 |
| *PK* | | | |
| Temperature | 1 | 57.250 | <0.001 |
| Supplementation | 2 | 3.833 | 0.041 |
| Temp * Suppl | 2 | 3.832 | 0.041 |
| *LDH* | | | |
| Temperature | 1 | 104.000 | <0.001 |
| Supplementation | 2 | 0.803 | 0.463 |
| Temp * Suppl | 2 | 3.671 | 0.046 |
| *FBP* | | | |
| Temperature | 1 | 14.510 | 0.001 |
| Supplementation | 2 | 0.006 | 0.946 |
| Temp * Suppl | 2 | 0.193 | 0.823 |
| ***Lipids metabolism*** | | | |
| *HADH* | | | |
| Temperature | 1 | 32.530 | <0.001 |
| Supplementation | 2 | 1.947 | 0.172 |
| Temp * Suppl | 2 | 6.225 | 0.001 |
| *GPDH* | | | |
| Temperature | 1 | 5.088 | 0.037 |
| Supplementation | 2 | 0.037 | 0.963 |
| Temp * Suppl | 2 | 1.549 | 0.239 |
| ***Amino acids metabolism*** | | | |
| *ALT* | | | |
| Temperature | 1 | 28.090 | <0.001 |
| Supplementation | 2 | 6.581 | 0.007 |
| Temp * Suppl | 2 | 2.406 | 0.119 |
| *AST* | | | |
| Temperature | 1 | 71.490 | <0.001 |
| Supplementation | 2 | 0.550 | 0.587 |
| Temp * Suppl | 2 | 1.602 | 0.229 |
| *GLDH* | | | |
| Temperature | 1 | 141.800 | <0.001 |
| Supplementation | 2 | 11.000 | 0.001 |
| Temp * Suppl | 2 | 5.346 | 0.015 |
